# Core clinical procedures and efficacy-enhancing strategies in boron neutron capture therapy

**DOI:** 10.1007/s12672-026-04774-y

**Published:** 2026-03-11

**Authors:** Qinqin Ma, Pengcheng Zhang, Huanyu Zhang, Hongxin Su, Tanglong Zhang, Zhuoya Zhang, Yuan Pan, Ruiming Chen, Juntao Ran

**Affiliations:** 1 The First Clinical Medical College of Lanzhou University, No. 199 Donggang West Road, Chengguan District, Lanzhou, 730000 Gansu China; 2 The First Hospital of Lanzhou University, No. 1 Donggang West Road, Chengguan District, Lanzhou, 730000 Gansu China

**Keywords:** BNCT, Boron delivery agents, Treatment planning system, Dynamic distribution, Combination therapy

## Abstract

Boron Neutron Capture Therapy (BNCT) is a binary targeted radiotherapy modality based on nuclear capture reactions. This technique exploits the tumor-targeting capability of boron compounds and their high thermal neutron capture cross-section, inducing localized nuclear reactions within cancer cells that generate α particles and lithium ions. This process enables selective tumor cell destruction at the cellular level. Clinical evidence demonstrates significant therapeutic efficacy of BNCT in treatment-refractory malignancies including glioblastoma, recurrent head and neck carcinomas, and cutaneous melanoma. Compared to conventional radiotherapy, BNCT leverages its inherent biological selectivity to achieve precise eradication of geometrically complex tumors and microscopic metastases, demonstrating significant clinical potential. However, the widespread adoption of BNCT remains constrained by several limitations, most notably the inadequate tumor selectivity of boron delivery agents. This review examines the literature published since the emergence of BNCT clinical research in 1970 up to the present, summarizing key clinical practices and strategies to enhance therapeutic efficacy, including boron carriers, administration regimens, treatment planning systems, real-time boron monitoring, and combination therapies. It aims to provide guidance for the clinical application of BNCT and support its broader adoption in practice.

## Introduction

 Neutron capture therapy (NCT), a form of targeted radiotherapy, operates through a biphasic mechanism [1]. In the initial phase, tumor-specific delivery of a stable isotope is achieved. The subsequent phase employs low-energy neutron irradiation to trigger isotope-specific nuclear reactions, producing high-LET particles and γ-rays that induce precise cytotoxic effects within the irradiated tissue. NCT primarily exists in two forms: Boron Neutron Capture Therapy (BNCT) and Gadolinium Neutron Capture Therapy (GdNCT), each utilizing distinct isotopes with unique neutron capture reactions and emission spectra [2]. However, the clinical application of GdNCT has been limited due to gadolinium’s high toxicity, poor targeting specificity, and the release of high-energy gamma rays during neutron capture reactions, which compromise therapeutic precision [3]. Consequently, BNCT remains the predominant clinical modality for treating malignant tumors.

**Fig. 1:**
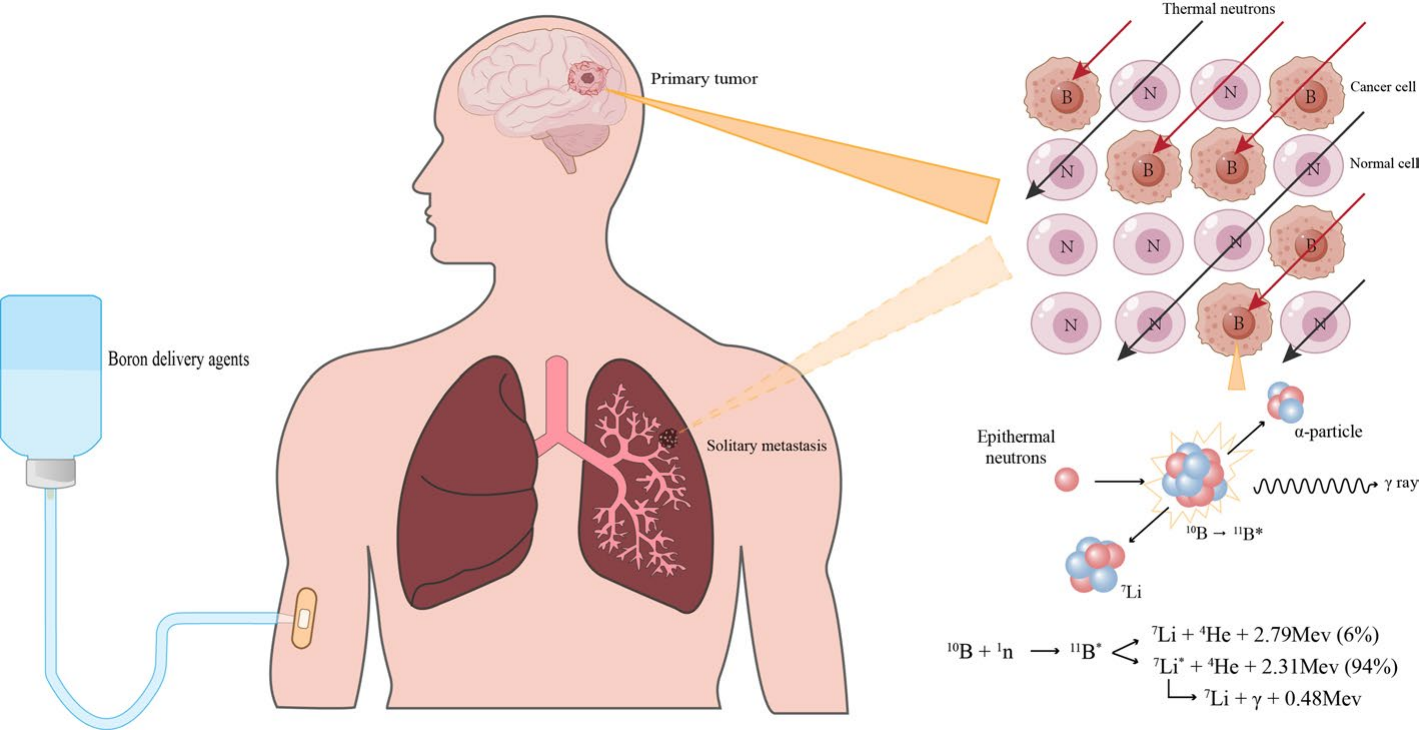
Schematic illustration of boron neutron capture therapy principle. Boron drugs are first administered intravenously, enabling targeted accumulation of ¹⁰B within tumor cells. Subsequent irradiation with thermal neutrons triggers the ¹⁰B (n, α) ⁷Li capture reaction, generating high-LET α and ^7^Li particles. This process results in the selective destruction of tumor cells while sparing adjacent healthy tissues. In addition to treating primary tumors such as glioblastoma, BNCT offers a viable therapeutic option for unresectable solitary metastases (e.g., lung metastases originating from colorectal cancer [7]). The dotted line indicates that these are distinct clinical scenarios and does not imply simultaneous irradiation of both sites

BNCT is a binary molecularly targeted particle therapy that requires the simultaneous presence of boron-containing drugs and neutron beams to achieve therapeutic effects. This process involves two stages: First, a boron compound containing the stable isotope ^10^B is administered to the patient, enabling ^10^B to selectively accumulate in tumor cells. Subsequently, irradiation with thermal or epithermal neutrons triggers the ^10^B (n, α)^7^Li nuclear reaction, releasing high-LET α particles and ^7^Li particles that destroy tumor cells [4, 5]. By synergistically combining the biological targeting of boron agents with the physical targeting of neutron beams, BNCT demonstrates unique advantages in treating diffuse or irregularly shaped solid tumors, as well as undetectable micro-metastases and subclinical lesions [6]. A schematic diagram of this mechanism is provided below (Fig. 1):

The α particles carry 1.47 MeV of energy, accounting for 63.6% of the total energy, while the ^7^Li ions carry 0.84 MeV, representing 36.4% [8]. Approximately 6% of these nuclear reactions produce ^7^Li in its ground state without gamma-ray emission, whereas 94% generate excited-state ^7^Li*, which subsequently releases a 0.48 MeV gamma ray during de-excitation [9]. Although prompt γ rays are emitted during BNCT, their energy is lower than the 511 keV annihilation gamma rays produced in positron emission tomography (PET) [10]. Thermal neutrons penetrate tissues without causing significant damage until captured by stable nuclei such as ^1^H, ^14^N, or ^10^B. Despite the ubiquitous presence of ^1^H and ^14^N in biological tissues, their neutron capture probabilities are comparatively low [11]. The capture cross-section—a measure of neutron absorption probability—is quantified in barns: ^1^H and ^14^N exhibit cross-sections of 0.33 barns and 1.7 barns, respectively, while ^10^B possesses an exceptionally high cross-section of 3,990 barns [12]. This stark contrast ensures that thermal neutrons are predominantly captured by tumor-localized ^10^B, thereby minimizing collateral damage to healthy tissues.

BNCT has been primarily limited by the challenges of neutron beam generation and the targeting specificity of boron-containing drugs. In recent years, with the continuous advancement of accelerator technology, it has become feasible to produce high-flux, stable neutron beams suitable for routine clinical use in BNCT [13, 14]. However, critical issues remain unresolved, such as the insufficient targeting capability of boron drugs, heterogeneous distribution of boron delivery agents within tumors, and tumor recurrence following BNCT. This review summarizes clinical procedures and efficacy-enhancing strategies for BNCT, aiming to facilitate its clinical translation and promote standardized implementation.

## Development of boron delivery agents

A critical aspect of BNCT lies in the application of boron-containing drugs. The primary boron agents currently used in BNCT are BPA (boronophenylalanine) and BSH (sodium borocaptate). However, BPA suffers from low boron content, necessitating repeated administration, while BSH exhibits poor tumor selectivity and often induces nonspecific tissue damage [15]. These limitations underscore the urgent need for novel boron carriers. An ideal boron delivery agent should meet the following criteria [8, 16, 17]:

1.  Good biocompatibility and low systemic toxicity.
2.  Sustain high tumor accumulation (20–50μg 10B/g tissue) with homogeneous intratumoral distribution.
3.  Enhance tumor targeting specificity, achieving a tumor-to-normal tissue boron concentration ratio (T/N ratio) > 3.
4.  Prolong tumor retention coupled with rapid systemic clearance.
5.  Cost-effectiveness, synthetic accessibility, and feasible clinical transformation.

The main goal of the new generation of boron delivery agents is to improve tumor targeting and boron loading, and most of them are boron delivery agents formed by chelating various targeted molecules and boron-containing compounds. Recently, Hong Xu et al. of China Pharmaceutical University have classified them based on the design strategy of boron delivery agents, on the one hand, it is a targeted strategy for the characteristics of tumor cells, compared with normal cells, tumor cell metabolism is more dependent on glycolysis, and the rate of value-added is also faster, so the demand for essential nutrients such as amino acids, glucose, and peptides increases, and cell membrane targeting can be carried out through L-type amino acid transport receptors (LATs), glucose transporters (GLUTs), peptides, and other molecules; BNCT mainly attacks DNA double-strands, so the nucleus is undoubtedly the best target for nuclear targeting through the use of nucleic acid derivatives and DNA intercalants [18]. On the other hand, there is a targeting strategy based on the properties of the compound itself, by modifying the structure and properties of boron carriers, such as applying liposomes, polymers, and inorganic nanoparticles to enhance the permeation and retention (EPR) effect, so as to improve its tumor targeting ability [19]. In addition, multiple targeting mechanisms such as porphyrins, which combine receptor-mediated active targeting and EPR effects, can be used to increase the content of drugs in tumors [20]. In the past few decades, various types of boron carriers have emerged in an endless stream, and many very promising new boron carriers have emerged, but the development of boron carriers has been limited due to a series of problems such as insufficient boron content, long metabolism time of macromolecular drugs, and difficulty in synthesis and preparation [21]. At present, there is still an urgent need for new boron carriers that can be efficiently applied in the clinical treatment of BNCT to help patients.

## Optimization of administration protocols

For BNCT to truly enter clinical practice, parameters such as drug dosage, administration regimen, and radiation dose must be standardized. However, different boron delivery agents may exhibit significantly distinct pharmacokinetic and pharmacodynamic profiles in tumor and normal tissues. This necessitates the evaluation of compound biodistribution characteristics through in vitro and in vivo experiments, while also optimizing the administration time window to ensure that the boron concentration in the tumor reaches the therapeutic threshold during neutron irradiation. This article summarizes the current administration regimens of commonly used boron drugs, aiming to support the clinical translation of boron delivery agents.

### BPA administration protocols

BPA, a structural analog of the amino acid phenylalanine, is transported into cells via LAT-1 (L-type amino acid transporter 1). Due to the upregulated amino acid transport characteristic of tumor cells, and because LAT-1 is highly expressed in many tumors such as head and neck squamous cell carcinoma and soft tissue sarcoma, BPA can accumulate within these tumor cells [22, 23]. This results in a degree of selective targeting of tumor cells by BPA. From 1998 to 2022, it was clinically used for brain and central nervous system tumors, and the dosage of BPA ranged from 290 to 900 mg/kg [24–26]; for head and neck tumors, BPA was administered at a total dose of either 400 mg/kg or 500 mg/kg [27, 28]; for skin cancer, the BPA dose ranges from 160 to 500 mg/kg [29–31]. It has also been used for other tumors such as extramammary Paget’s disease and spindle cell carcinoma, and the BPA dose ranges from 200 to 500 mg/kg [32, 33]. In addition to variations in administration dosage, the routes of BPA delivery also differ significantly, including peritumoral administration, intravenous injection, and gavage administration [34–36]. A two-stage BPA infusion protocol—200 mg/kg/h for 2 h pre-BNCT, followed by 100 mg/kg/h for 1 h during neutron irradiation—is now widely adopted for head and neck cancer. This regimen ensures sustained intratumoral boron accumulation during irradiation, yielding a total BPA dose of 500 mg/kg [28, 37]. This two-phase infusion ensures stable blood boron concentration during neutron irradiation. By employing inductively coupled plasma atomic emission spectroscopy (ICP-AES), the boron concentration can be quantified 2 h post-infusion, enabling real-time calculation of intratumoral boron levels during irradiation [38]. This pharmacokinetically guided approach allows predictive assessment of BNCT therapeutic efficacy.

Following intravenous administration, BPA undergoes biphasic elimination: the first stage is rapid distribution phase, which refers to BPA rapidly partitions from blood into tumors and peripheral tissues, with a half-life (t_1/2_) of 0.7–3.7 h [13]. The second stage, the slow clearance phase, refers to BPA gradually redistributes from tissues back to systemic circulation for renal excretion, characterized by a prolonged terminal half-life (t_1/2_) of 7.2–12.0 h [13]. The biphasic elimination kinetics of BPA guide its clinical application by defining the optimal irradiation window. Neutron exposure is typically initiated 2 h post-infusion, when the tumor-to-normal tissue boron concentration ratio (T/N ratio) reaches its peak value. Furthermore, patients with renal insufficiency require protocol adjustments—either dose reduction or extended infusion duration —to mitigate systemic accumulation risks.

### BSH administration protocol

The uptake of BSH primarily depends on passive diffusion and is particularly dependent on the disruption of the blood-brain barrier (BBB) in tumor regions. Normal brain tissue with an intact BBB exhibits minimal BSH uptake. Therefore, BSH is predominantly utilized for central nervous system tumors, such as gliomas and meningiomas, where the BBB is compromised [39]. Although BSH exhibits inferior tumor targeting compared to active transport mechanism of BPA, its 12-fold higher boron payload enables significant dose reduction. Between 1994 and 2008, BSH was clinically administered for brain and central nervous system tumors at doses ranging from 30 to 100 mg/kg [40–42]. Based on findings from the EORTC 11,961 trial, the optimal regimen was established as 100 mg/kg administered at an infusion rate of 1 mg/kg/min, demonstrating both safety and efficacy [15]. This protocol has become the clinical standard, with neutron irradiation typically initiated 12–14 h post-infusion, corresponding to a tumor-to-blood boron concentration ratio (T/B ratio) of 0.6 ± 0.2 [43]. However, the heterogeneous intratumoral boron distribution following BSH administration renders precise calculation of tumor-absorbed dose impractical. Consequently, radiotherapy planning prioritizes adherence to normal brain tissue tolerance doses rather than tumor-specific dosimetry.

## Treatment planning system

**Fig. 2:**
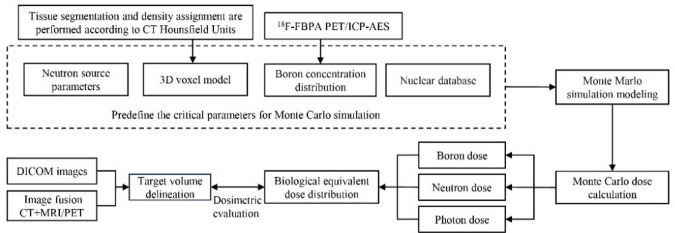
Workflow of a BNCT treatment planning system

The successful implementation of BNCT relies critically on its Treatment Planning System (TPS), which differs significantly from those used in conventional photon radiotherapy. BNCT requires Monte Carlo (MC) modeling to accurately simulate the mixed radiation field—comprising neutrons, photons, and other particles—produced during irradiation [44]. The TPS converts CT images into a three-dimensional voxel-based model, assigning specific biological materials (such as muscle, bone, air, and water) based on CT Hounsfield values. Boron distribution, estimated from PET (Positron Emission Tomography) or T/N ratios measured by ICP-AES (Inductively Coupled Plasma Atomic Emission Spectrometry), is mapped onto this anatomical model [45, 46]. Using neutron source parameters, nuclear reaction cross-section data, and the 3D voxel model, the MC method simulates the BNCT process, calculating the physical dose (D) contributions from boron capture, nitrogen capture, hydrogen scattering, and gamma rays. These physical doses are then converted into biological equivalent doses (BED) using biological weighting factors [47]. Finally, the system generates a dose distribution overlay onto the patient’s CT or MRI images, allowing visual evaluation of dose in both target and normal tissues. The entire workflow of the BNCT planning system is shown in Fig. 2.

While general-purpose MC engines such as MCNP are commonly used—which reduces the development difficulty of TPS—their powerful and broad applicability necessitates extensive cross-section libraries and subroutines, leading to computationally cumbersome and slow processes. Furthermore, they often lack the quality control and testing requirements specific to medical software. To address these limitations, Wan Bing Zhong et al. developed Compact Particle Simulation System (COMPASS), a dedicated dose calculation engine for BNCT [48]. This system achieves a significant reduction in computation time without compromising dose calculation accuracy. This advancement has facilitated continuous optimization of treatment planning systems, from SERA to NeuCure and CICS, and then to NeuMANTA—all evolving toward greater precision, simplification, and efficiency to expand the clinical application of BNCT [45, 49, 50].

## Dynamic monitoring and personalized drug delivery

BNCT relies on the precise accumulation of ^10^B in tumor tissue, making accurate monitoring of boron distribution critical to its success. However, current clinical practice primarily involves measuring blood boron concentration via ICP-AES before irradiation to estimate intratumoral boron levels during treatment [51]. This approach contradicts the principle of precision therapy in BNCT, as boron distribution in tumor tissue is inherently heterogeneous. Therefore, the application of imaging technology is essential. First, it enables real-time monitoring of boron distribution to ensure that the tumor region reaches the therapeutic boron concentration threshold [52]. This clarifies the difference in boron distribution between tumor and normal tissues, optimizes the irradiation field of the neutron beam, and maximizes the protection of healthy tissues. Second, boron uptake may vary among patients, and even within the same patient, boron concentrations can fluctuate during treatment and imaging facilitates personalized adjustments to irradiation doses [53]. Additionally, as the treatment process may require multiple irradiations, imaging can track changes in boron concentration to ensure the efficacy of each session. It also provides imaging-based evaluation of tumor shrinkage, guiding subsequent treatment steps.

Comparison with SPECT (Single Photon Emission Computed Tomography), PET exhibits advantages such as high sensitivity, precise quantification, metabolic imaging, and multi-modal fusion. Therefore, PET is more widely utilized in BNCT imaging [54, 55]. Currently, typically performed one week prior to the intended treatment, ¹⁸F-FBPA PET imaging is used for clinical patient selection and treatment planning in BNCT. Following intravenous administration of the tracer, scans are acquired 20 to 60 min later to assess boron agent biodistribution [51, 56]. Manually delineated regions of interest (ROIs) are drawn on tumor lesions to calculate the maximum standardized uptake value (SUV_max_), reflecting radiotracer uptake intensity in the most metabolically active tumor areas. For specific organs/normal tissues, volumes of interest (VOIs) are contoured to obtain total activity and mean standardized uptake value (SUV_mean_) within the defined volumes [57]. The T/N and T/B ratios are defined as the SUV_max_ of the tumor divided by the SUV_mean_ of normal tissues and blood, respectively, following ^18^F-BPA administration, to evaluate the tumor targeting capability of boron agents and provide critical evidence for personalized BNCT treatment [56, 57].

$$ \frac{T}{N} = \frac{{SUVmax~of~the~tumor~region}}{{SUVmean~of~normal~tissue}} $$

**Fig. 3:**
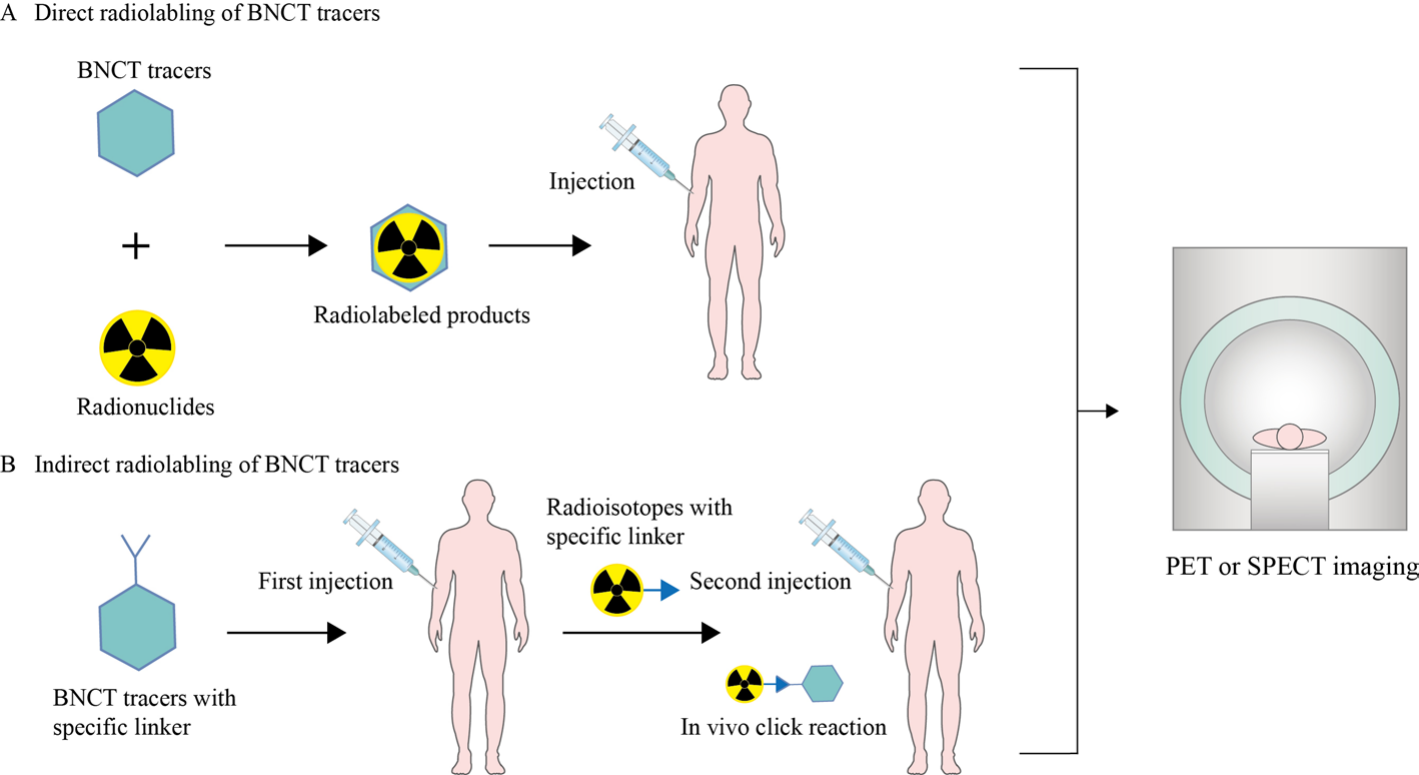
Schematic illustration of radiolabeling strategies for molecular imaging. **A** Direct radiolabeling of BNCT tracers. **B** Indirect radiolabeling of BNCT tracers

Due to the prolonged drug infusion time required in BNCT to achieve stable blood boron concentration during irradiation, and the short half-life of ¹⁸F (110 min), administration prior to treatment would result in its decay to undetectable levels within the several-hour treatment window. This precludes its use for real-time monitoring and dose adjustment. Furthermore, the currently clinically used ¹⁸F-FBPA suffers from limitations such as insufficient tumor targeting and metabolic instability. Consequently, numerous novel BNCT imaging agents have been developed in recent years. Park Ji Ae and colleagues comprehensively summarized BNCT tracers, categorizing them into two classes based on their radionuclide labeling strategies [58]. As shown in Fig. 3, the one type is direct labeling, where a boron delivery agent directly reacts with a radionuclide to form a radioactive boron delivery agent for imaging. The other type is indirect labeling, where a boron delivery agent with a specific linker is first injected into the body. When it reaches maximum accumulation in tumors and is largely cleared from healthy tissues, a radionuclide with a complementary linker is administered. The two components undergo an in vivo “click reaction” to enable imaging. As presented in Table 1, this paper lists promising PET-based BNCT tracers, summarizes their T/N ratios, advantages, and limitations, and provides insights to promote the integration of diagnosis and treatment in BNCT, achieving the goal of precision treatment.

**Table 1:** Essential features of radiolabeled BNCT tracers for PET imaging

Tracer	T/*N* ration	Maximum uptake value of 10B in tumors	Tumor type	Advantages and Application Prospects	Limitations and Potential Improvements	References
^18^F-FBPA	3.5 ± 0.24	28.48 ± 6.4%ID/g	Head and Neck Squamous Cell Carcinoma	Most clinically established	Short half-life of ^18^F, metabolic instability and complex synthesis process	[59]
^18^F-FBY	24.56 ± 6.32; 2.30 ± 1.26	2.84 ± 0.46 ppm; 0.28 ± 0.14ppm	High-Grade Glioma; Low-Grade Glioma	High stability, high water solubility, and validated in human studies	Lack of large-scale cohort studies	[60]
^18^F-BBPA	18.7 ± 5.5	4.21 ± 0.30%ID/g	Malignant Brain Tumor	High Tumor Uptake and boron content	Synthetic Complexity	[61]
^64^Cu-BSH-3R-DOTA	15.5	2.25%ID/cc	Malignant Glioma	Cost effective and simple synthesis process	Lack of clinical validation	[62]
^64^Cu-BPN	T/M and T/B rations were 61.46 ± 20.26 and 33.85 ± 5.73, respectively	125.17 ± 13.54 ppm	Melanoma	High tumor accumulation	premature release of the boron drug from micelles before reaching the tumor site	[63]
^64^Cu-DSPE-BCOP-5T	T/M and T/B ratios were 25.20 ± 3.41 and 7.46 ± 0.66, respectively	84.93 ± 2.68 ppm	Breast Cancer	Good micellar stability, tunable molecular size, and high drug loading efficiency	Lack of clinical validation	[64]
^64^Cu-AuNP-TCO-COSAN	Not mentioned	4.76 ± 1.85%ID/cm^3^	Breast Cancer	Strong tumor targeting specificity	synthetic complexity due to multi-component integration	[65]
^64^Cu-Tz-B-AuNRs	T/M ration was 5.4 ± 2.7	3.3 ± 1.2%ID/cm3	Gastric Adenocarcinoma	AuNRs load with a high boron payload and can combined photothermal therapy	Low boron accumulation in tumors; technical challenges in combining two treatment modalities	[66]
^64^Cu-NOTA-boronsome	T/M and T/F rations were 36.9 ±1.4 and 26.6 ± 1.5, respectively	5.49 ± 0.22%ID/g or 93.3 ppm	Breast Cancer	Combination with chemotherapy, such an encapsulated PARP1 inhibitors, may enhance efficacy	Synthetic complexity and lack of human validation	[67]
^64^Cu-PTL @BNNPs	T/B, T/M and T/F rations were2.71 ± 0.96, 8.32 ± 1.07 and 5.96 ± 0.37	6.18 ± 2.18%ID/g	Triple Negative Breast Cance	Vitamin C triggered degradation reduces side effect	Lack of clinical validation	[68]
^68^Ga-PSMA-11	T/M ration was 2	4–7 ug/g	Prostate Cancer	Boron-PSMA therapy for prostate cancer is feasible with further optimization	low tumor uptake currently insufficient for clinical application	[69]
^68^Ga-DOTA-c(RGDFK)	Not mentioned	4–6%ID/g	Glioblastoma	^68^Ga is readily accessible; high tumor uptake was observed	further irradiation experiments have not been conducted	[70]

T: tumor; B: blood; F: fat; M:muscle; %ID/g: percentage of injected dose per gram of tissue; FBY: fluorinated boron tyrosine; BBPA: p-boronophenylalanine; DOTA: 1,4,7,10-tetraazacyclododecane-1,4,7,10-tetraacetic acid; BPN: Boron-loaded polymeric nanoparticles; DSPE: 1,2-distearoyl-sn-glycero-3-phosphoethanolamine; BCOP: boron cluster-conjugated oligopeptide; 5T: pentapeptide; AuNP: gold nanoparticles; TCO: trans-cyclooctene; COSAN: [3,3’-Co(1,2-C₂B₉H₁₁)₂]⁻; Tz-B: tetrazine-boron conjugate; AuNRs: gold nanorods; NOTA: 1,4,7-triazacyclononane-1,4,7-triacetic acid; PTL@BNNPs: paclitaxel-loaded boron nitride nanosheets; PSMA-11: prostate-specific membrane antigen ligand-11; c(RGDFK): cyclic(Arg-Gly-Asp-D-Phe-Lys)

## BNCT combination therapy

While BNCT has demonstrated unique targeting advantages in the treatment of certain malignant tumors, with clinical trial data indicating an objective response rate (ORR) of 60–80% post-BNCT, tumor recurrence remains a critical unresolved challenge [28, 71]. A clinical study from the Fukushima BNCT Clinical Research Center in Japan revealed that patients with locally advanced head and neck cancer treated with BNCT exhibited a local recurrence rate as high as 33% within 1 year [72]. This recurrence tendency is closely associated with inadequate boron targeting, heterogeneous drug distribution, and dosimetric constraints of neutron beams. To overcome the limitations of monotherapy and prevent post-BNCT tumor recurrence, current international research advocates multimodal combination therapy strategies.

### BNCT Combined with Irradiation

BNCT demonstrates clinically favorable response rates with acceptable toxicity profiles. However, monotherapy limitations persist due to the absence of ideal boron delivery agents, manifested in three key constraints: heterogeneous intratumoral distribution of boron delivery agents, inadequate clinical target volume (CTV) coverage, and compromised neutron penetration for deep-seated tumors [73]. Combining BNCT with complementary radiotherapy modalities addresses these deficiencies by improving dose homogeneity, enhancing CTV irradiation, and enabling elective nodal irradiation for high-risk metastases. Current protocols typically administer photon therapy 2–4 weeks post-BNCT after resolution of acute inflammatory reactions (e.g., edema, erythema).

A phase II trial investigating BNCT combined with image-guided intensity-modulated radiotherapy (IG-IMRT) revealed no significant improvement in local progression-free survival (PFS) or overall survival (OS) compared to BNCT alone [71]. Notably, tumors exceeding 100 cc volume demonstrated increased susceptibility to grade 4 toxicities under combination therapy due to extended irradiation volumes. Subsequent investigations explored BNCT coupled with carbon ion radiotherapy (CIRT), achieving optimal tumor control with minimal normal tissue toxicity—particularly effective for large, polymorphic, radioresistant, and infiltrative neoplasms [74]. For small superficial tumors, however, combination strategies confer limited advantage over BNCT monotherapy. While BNCT demonstrates compatibility with photons, protons, and heavy ions, current evidence remains insufficient to establish therapeutic superiority of any specific combination. Clinical implementation requires careful consideration of tumor volume, depth, invasiveness, and patient tolerance. Treatment selection must be individualized through customized dose optimization to balance locoregional control against potential toxicities.

### BNCT combined with chemotherapy

Combination therapy is widely employed in oncology to enhance therapeutic efficacy and improve prognosis. Chemotherapeutic agents can modulate the tumor microenvironment, increase BBB permeability to promote intratumoral boron accumulation, and potentiate BNCT-induced tumor cell death by interfering with DNA repair mechanisms or arresting tumor cells in radiation-sensitive phases of the cell cycle. To optimize the synergistic antitumor effects of chemotherapy, boron nitride nanosheets (BNNSs) were engineered as a dual-functional drug delivery system, co-loading ^10^B and doxorubicin (DOX) to form DOX@BNNSs [75]. Their study demonstrated that neutron irradiation triggered DOX release at tumor sites, enhancing in vivo antitumor efficacy. Notably, the tumor boron uptake and tumor-to-blood (T/B) ratio of DOX@BNNSs surpassed those of BPA, indicating superior tumor-targeting specificity. Temozolomide (TMZ), a cornerstone drug for glioblastoma (GBM) via DNA alkylation and BBB penetration, was modified by Jing Xiang et al. to create a ^10^B-labeled derivative, TMZB [76]. In TMZB-treated groups, both intratumoral boron concentration and T/B ratios were significantly higher than those with BPA, confirming enhanced tumor-targeted boron delivery. Importantly, TMZB retained its inherent cytotoxicity against GBM while synergizing with BNCT. These studies highlight that integrating chemotherapeutic agents with boron carriers not only amplifies their anticancer effects but also achieves remarkable therapeutic synergy with BNCT, demonstrating promising clinical potential.

### BNCT combined with immunotherapy

Immunotherapy has demonstrated efficacy against various cancers by activating systemic immune responses to suppress both primary and metastatic lesions. Its durable therapeutic effects are driving a paradigm shift in oncology, transforming cancer from a “terminal disease” to a manageable chronic condition. Preclinical studies have demonstrated that BNCT exhibits significant immunostimulatory properties when combined with immunomodulatory agents. Specifically, in colorectal cancer models, the combination of BNCT and Bacillus Calmette-Guérin (BCG) enhanced antitumor immunity, while in drug-resistant melanoma models, BNCT synergized with immune checkpoint inhibitors (ICIs) to significantly suppress tumor growth [77, 78]. This immunomodulation stems from BNCT-induced immunogenic cell death (ICD), which triggers the release of tumor-associated antigens, consequently priming immune activation and potentiating the efficacy of ICIs [79]. Furthermore, combination therapy amplifies the abscopal effect, generating systemic antitumor responses against non-irradiated metastases [80]. Although preclinical studies have confirmed the feasibility and enhanced antitumor activity of BNCT in combination with immunotherapy, several critical questions remain unresolved: which specific effector cell populations mediate these responses, what tumor-immune mechanisms are activated, whether this combination can be translated into clinical trials, and what constitutes the optimal immunotherapy regimen for use with BNCT [81]. Addressing these questions will be essential for translating this promising strategy into future clinical applications.

### BNCT combined with other therapeutic approaches

Ultrasound is widely utilized in disease screening, diagnosis, and treatment due to its real-time dynamic imaging capabilities, high safety profile, and multifunctionality. Critically, ultrasound can enhance drug delivery and reduce cell viability through thermal and mechanical effects. Previous studies have confirmed that high-intensity focused ultrasound (HIFU) followed by intravenous boron agent administration or co-delivery of boron agents with ultrasound microbubbles (MBs) increases ^10^B concentration at tumor sites, thereby amplifying tumor cell death [82, 83]. Furthermore, post-BNCT HIFU application targeting the gross tumor volume (GTV) and tumor margins can further eradicate residual cancer cells via thermal ablation and mechanical disruption, compensating for heterogeneous boron distribution and improving BNCT efficacy [84].

Electroporation is a physical technique that utilizes transient, high-intensity electric fields to temporarily increase cell membrane permeability, thereby significantly enhancing the intracellular uptake of therapeutic agents. In oncology, electroporation has been employed to facilitate the delivery of chemotherapeutic drugs or gene therapy vectors, demonstrating favorable efficacy and safety [85, 86]. Recently, combining electroporation with boron neutron capture therapy has shown promise in preclinical studies. Electroporation boosts tumor cell accumulation of boron agents, thereby improving boron biodistribution and BNCT targeting [87]. This strategy may enhance local tumor control, particularly in deep-seated tumors, while reducing normal tissue damage through selective boron uptake [88]. Given the established clinical application of electroporation, this provides a practical foundation for its combination with BNCT. Therefore, this synergistic approach holds significant therapeutic promise and potential for clinical translation.

BNCT destroys tumor cells by triggering a nuclear reaction between ^10^B and neutrons within the tumor cells. Unlike conventional radiation therapy, which relies on the oxygen effect to kill cells, BNCT remains effective against hypoxic tumor cells. However, studies have confirmed that the uptake of boron compounds is limited in hypoxic and quiescent cell populations [89]. Furthermore, since BPA is actively taken up via the LAT-1 transporter protein, hypoxia has a more significant impact on BPA uptake [90]. Yuki Wada et al. found that in human glioblastoma, BPA uptake gradually decreases as oxygen concentration declines, without a critical threshold [91]. This is primarily because lat-1mRNA expression is down-regulated under hypoxic conditions, leading to reduced boron uptake and consequently diminished BNCT efficacy [91]. Therefore, maintaining local oxygen concentration within the tumor may be a promising approach to enhance BNCT efficacy and reduce tumor recurrence rates after BNCT treatment.

## Discussion

This paper first discusses the theoretical advantages of BNCT, which achieves tumor cell-level destruction through a binary molecular targeting mechanism, and validates its safety profile with concrete data. Clinical studies have confirmed BNCT’s efficacy in treating head and neck cancers, gliomas, and other malignancies. However, challenges such as suboptimal boron agent targeting, heterogeneous boron distribution, and post-BNCT tumor recurrence persist, limiting its broader clinical adoption [92]. The most direct approach to addressing the insufficient tumor-targeting specificity of current boron agents is the development of novel boron compounds. Over the past decade, a wide array of boron-based agents had emerged, demonstrating encouraging results in preclinical studies. However, their clinical translation has been hindered by limitations such as inadequate boron payload, synthetic complexity, and prolonged metabolic retention [21]. At present, second-generation boron agents—BPA and BSH—remain the mainstay in clinical practice. Optimizing their administration protocols, enhance boron utilization efficiency, and establishing standardized treatment guidelines, could facilitate the clinical adoption of these agents. Furthermore, due to the multiple types of radiation involved in BNCT, its treatment planning system requires the implementation of MC simulations to precisely calculate the energy deposition in tissues during irradiation. Due to interpatient variability in boron agent uptake and metabolism, accurate detection of boron distribution in tissues is essential to screen out patients with inadequate T/N ratios, predict therapeutic efficacy, and enable real-time adjustments of neutron irradiation parameters [93]. Finally, combination therapeutic modalities were proposed to mitigate post-BNCT recurrence, multimodal strategies integrating spatial targeting precision, molecular specificity, and immune modulation are emerging as solutions to overcome monotherapy limitations.

In recent years, the rapid development of accelerator-based neutron sources has reignited interest in BNCT, which offers significant advantages over conventional radiotherapy for locally invasive tumors. This momentum is reflected in the inclusion of accelerator-based BNCT for recurrent head and neck cancer in Japan’s national health insurance scheme in 2020 [94], as well as the ongoing expansion of preclinical and clinical studies targeting brain tumors, liver cancer, pancreatic cancer, breast cancer, skin tumors, thoracic malignancies, and urological tumors [69, 95–100]. Notably, real-world data from the first two years of insurance-covered treatment (69 patients) have confirmed an objective response rate of 80.5% with manageable toxicity, supporting the feasibility of BNCT in routine clinical practice [101]. Despite these advances, BNCT still faces several limitations, including the lack of more precise boron delivery agents, inaccuracies in radiation dosimetry, restricted indications, and the risk of post-treatment tumor recurrence. Only by overcoming these critical obstacles can BNCT evolve from an attractive theoretical concept into a genuinely effective clinical treatment modality.

## Data Availability

No datasets were generated or analysed during the current study.
